# Genome analysis of *Ceratobasidium theobromae* and its causal association with cassava witches’ broom disease in the Philippines

**DOI:** 10.3389/ffunb.2026.1800255

**Published:** 2026-04-17

**Authors:** Cris Q. Cortaga, Alejandra Gil-Ordóñez, Mónica F. Fronda, John Emmanuel B. Tandang, Juan M. Pardo, Ana M. Leiva, Warren Arinaitwe, Clarise Marie S. Evaristo, Jonathan Newby, Al Imran Malik, Wilmer J. Cuellar

**Affiliations:** 1 Plant Pathology Laboratory, Institute of Plant Breeding, College of Agriculture and Food Science, University of the Philippines Los Baños, College, Laguna, Philippines; 2 Crop Protection Research Area, Cassava Program, International Center for Tropical Agriculture (CIAT), Palmira, Colombia; 3 Departamento de Biología, Facultad de Ciencias Naturales y Exactas, Universidad del Valle, Cali, Colombia; 4 Cassava Program Asia-Office, International Center for Tropical Agriculture (CIAT), Vientiane, Lao People’s Democratic Republic; 5 Department of Agriculture, Kasetsart University (KU), Bangkok, Thailand

**Keywords:** fungal genome, genetic diversity, insular, Southeast Asia, *Theobroma cacao*

## Abstract

Cassava witches’ broom disease (CWBD) has been present in the Philippines from the early 2010s and has since been considered a phytoplasma-caused disease. This assumption has guided CWBD diagnostics and management, yielding mixed results. However, recent results in continental Southeast Asia (SEA) indicate that the disease is rather associated with the fungus *Ceratobasidium theobromae*, the causal agent of Vascular Streak Dieback (VSD) of cacao, another important disease occurring in the Philippines. Field surveys showed that CWBD was present in all cassava-growing regions, reaching field level incidence values above 50%. *C. theobromae* was detected in symptomatic samples using a standard PCR assay, with a sensitivity of 91.79% and a specificity of 95.24%, supporting its causal association with CWBD, in contrast to phytoplasma detection, which failed to yield positive results. Genome analyses of *C. theobromae*, supported by an improved reference genome, indicate that Philippine isolates, whether infecting cassava or cacao, are more closely related to each other than to isolates from continental SEA. Furthermore, isolates from the recent outbreak in the Americas are more closely related to those from continental SEA than to those from the Philippines.

## 1 Introduction

Cassava storage roots are the third most important source of calories in the Philippines, after rice and corn. However, cassava cultivation is continuously threatened by pests and diseases, such as cassava witches’ broom disease (CWBD), which remains a significant constraint to cassava production in the country (Dolores et al., 2023). Although farmers have long recognized CWBD symptoms, significant yield losses have only recently been reported due to the expansion of commercial cassava production in the country along with the disease. Initially spotted in the provinces of Eastern Visayas, the disease has eventually spread to the province of Bukidnon in Mindanao, where cassava is grown and commercialized on a large scale (Vasquez et al., 2016; Dolores et al., 2023). This prompted the release of a Special Quarantine Order in Bukidnon in 2015 by the Department of Agriculture-Bureau of Plant Industry to protect the cassava areas and control the disease spread to nearby provinces in the Philippines (Dolores et al., 2023).

Correct identification of a disease causal agent is essential to understand its epidemiology, enabling effective management and risk assessment. Although Koch’s postulates remain the classical framework for confirming causal agents (Ross and Woodward, 2016), their application becomes problematic when organisms cannot be cultured *in vitro* or fail to meet all criteria, particularly in the case of obligate parasites or fastidious microbes (Fox, 2020). This challenge is evident for candidate pathogens associated with CWBD. Historically, the disease in Southeast Asia (SEA) has been linked to insect-vectored phytoplasmas belonging to up to five different ribosomal groups reported in this region (Pardo et al., 2023), including the most recently ‘*Candidatus* Phytoplasma luffae’-related strain (16SrVIII) reported in the Philippines (Dolores et al., 2023). Aside from cassava, the latter phytoplasma strain has also been detected in loofah, bitter gourd, tomato, and bamboo (Dolores et al., 2023; Sta Cruz et al., 2021; Borines et al., 2020). However, a comprehensive assessment of their association with CWBD is still lacking. This gap is further complicated by the high incidence of false positives in phytoplasma detection using standard molecular diagnostics (Pardo et al., 2023). On the other hand, a recent study was able to show a strong association of CWBD with the fastidious fungus *Ceratobasidium theobromae* (syn. *Rhizoctonia theobromae*) in continental SEA (Leiva et al., 2023). This association has recently been confirmed in French Guiana and Brazil by different groups analyzing the recent outbreak of CWBD in the Americas (Pardo et al., 2024; Oliveira et al., 2025).

At present, the disease is being managed through cultural practices that call for additional inputs and cost in cassava cultivation, as well as the use of antibiotics to target the believed phytoplasma (bacterial) pathogen, or pesticides to control its putative insect vector (Landicho et al., 2024). However, the widespread use of these chemicals driven by the absence of a clearly associated pathogen and the lack of specific control strategies has raised concerns about its effectiveness and potential impact on environmental and plant health (Symochko et al., 2023). Moreover, the continued reliance on broad, non-specific treatments risks promoting antimicrobial resistance, disrupting beneficial microbial communities, and imposing further economic burdens on smallholder growers (Batuman et al., 2024). Ultimately, the uncertainty surrounding the etiological agent not only hampers the development of targeted management strategies but also perpetuates practices that may be unsustainable in the long term.

*C. theobromae* is a basidiomycetous fungus that has been considered pathogenic since the 1960s, when it was first linked to Vascular Streak Dieback (VSD) of cacao (Talbot and Keane, 1971**;** Keane et al., 1972). Biological characterization of this fungus show that its filamentous mycelia are typically yellowish to white, with hyphal walls 0.9 to 2 µm thick. Newly emerging hyphae branch at right angles, with branching points that are usually septate and slightly inflated, while the main hypha remains straight and hyaline with an average hyphal width of approximately 5 µm (Junaid and Guest, 2021). Like other fastidious fungi, *C. theobromae* is difficult to culture and maintain *in vitro*, as isolates often persist for only a few transfers and are frequently compromised by microbial contaminations, limiting the availability of pure cultures and high-quality genomic DNA for further studies.

Despite these constraints, a first *C. theobromae* draft genome—derived from infected cacao—was reported in 2019 (Ali et al., 2019), enabling comparisons with cassava isolates and confirming their taxonomic identity based on whole-genome data and additional genetic markers such as ITS, *rpb2*, *tef1*, and *atp6*. Nevertheless, most phylogenetic analyses have relied primarily on the ITS region (Samuels et al., 2012). Since the publication of diagnostic primers by Leiva et al. (2023), the calcium/calmodulin-dependent protein kinase gene (CAMK/CAMKL; hereinafter CAMK) has become the genomic region with the most extensive sequence representation of *C. theobromae* in NCBI GenBank. Annotated in the first *de novo* genome (GenBank accession KAB5596398), CAMK encodes a serine/threonine kinase containing conserved ATP- and substrate-binding motifs and a well-defined activation loop, and plays key roles in Ca²^+^-mediated signaling pathways involved in fungal growth, stress responses, and possibly virulence (Ding et al., 2014). The primers amplify a fragment corresponding to amino acids 394–745 of the predicted protein (nucleotides 1183–2235), a region that is highly conserved among isolates (>97% nucleotide identity) (Leiva et al., 2023; Pardo et al., 2024). Given its extensive representation, diagnostic specificity, and functional relevance, this marker provides a robust foundation for an initial assessment of genetic diversity across isolates from infected cassava and cacao, while enabling exploration of their geographical and temporal structure.

Here, we confirm the association of *C. theobromae* with characteristic symptoms of CWBD (such as vascular necrosis, proliferation of short petioles, leaf yellowing) in nine provinces of the Philippines. The genetic identity of the pathogen associated with CWBD in the Philippines was further confirmed by genome assembly and marker gene analysis, and including isolates from cacao. A global analysis of *C. theobromae* isolates also revealed that those from the recent CWBD outbreaks in the Americas are more closely related to isolates from continental SEA than to those from the Philippines, and form a genetically distinct lineage that has likely undergone recent expansion.

## 2 Materials and methods

### 2.1 Field surveys, sample collection, and symptom recording

Cassava-producing provinces were identified based on publications and production statistics in the Philippines. Among these provinces we organized field surveys in Isabela, Quezon, Camarines Sur, Samar, Leyte, Bohol, Cebu, Iloilo, and Bukidnon which were visited from March to September 2024. Petiole samples and stem cuttings from symptomatic and healthy cassava plants were collected, and photographic records were taken from 30 plants per field, collected along a diagonal transect every fourth plant. All data and metadata (**Supplementary Table 1**) are also available from our repository PestDisPlace (Cuellar et al., 2022). Petiole samples and stem cuttings collected were placed in dry tissue papers for moisture absorbance, secured in labelled plastic zip locks, and then brought to the Plant Pathology Laboratory of the Institute of Plant Breeding (IPB), University of the Philippines Los Baños (UPLB) for processing and DNA extraction. Representative infected stem cuttings were also planted in pots with sterile soil and maintained inside insect-proof cages for growing and further study.

### 2.2 DNA extraction and PCR-based diagnostics

DNA was extracted using the CTAB protocol (Jimenez et al., 2021) and adjusted to a working concentration of 30 ng/µl. DNA integrity was verified by internal control PCR using primers rbcLa-F and rbcLa-R target of ribulose bisphosphate carboxylase gene (*rbcLa*) (Kress and Erickson, 2007). PCR reactions were prepared using 1µl of DNA extracts, 2X GoTaq^®^ Master Mix Green (Promega, USA), 200 nmol of each primer and nuclease-free water in a 25 µl reaction volume. PCR tests were carried out in a Mastercycler^®^ Gradient Thermal cycler (Eppendorf, USA), under the following conditions: 95 °C for 5 min; 30 cycles of 95 °C for 30 s, 55 °C for 30 s, and 72 °C for 1 min; and a final extension at 72 °C for 10 min. Only samples producing an rbcLa-R PCR product were used for the association analyses.

Detection of *C. theobromae* was performed by PCR following the protocol designed by Leiva et al. (2023) which targets the CAMK gene. Other pathogens reported in the SEA region were also tested, including Sri Lankan cassava mosaic virus (SLCMV) and phytoplasma. Detection of SLCMV was performed by PCR following the protocol designed by Siriwan et al. (2020) which targets the coat protein gene of the virus. In addition, phytoplasma detection was conducted by nested PCR as previously reported (Dolores et al., 2023), with minor modifications. The first round employed universal primers P1/P7 (Deng and Hiruki, 1991; Schneider et al., 1995), and the second round used primers R16MF2/R1 targeting the 16S rRNA regions of ‘*Candidatus* Phytoplasma luffae’ (Gundersen and Lee, 1996). Reactions were prepared using GoTaq^®^ Flexi DNA Polymerase (Promega) in 25 µl volumes. Thermal cycling conditions for the first round consisted of 94 °C for 2 min; 35 cycles of 94 °C for 1 min, 50 °C for 1 min, and 72 °C for 2 min; and a final extension at 72 °C for 10 min. The second round followed the same profile, with an extension time of 1 min 30 s at 72 °C. All PCR products were visualized by electrophoresis on a 1% agarose gel.

Positive diagnostics for *C. theobromae* using CAMK primers were confirmed either through outsourced bidirectional Sanger Sequencing (Macrogen Inc., South Korea) or using the standard amplicon sequencing protocol with Oxford Nanopore Technologies (ONT) as it follows. PCR products were sequenced on a MinION Mk1B using the SQK-LSK114.24 kit following the manufacturer’s instructions and loaded onto an R10.4.1 flow cell (FLO-MIN114) for 72 h. Basecalling was performed using Dorado v0.5.3 with the high-accuracy model (hac). Amplicons were assembled *de novo* using Amplicon_sorter (Vierstraete and Braeckman, 2022) with default parameters, and coverage for each consensus sequence was assessed with Qualimap v2.2.2 (García-Alcalde et al., 2012). Only consensus sequences with coverage greater than 250X were included in downstream analysis.

### 2.3 Amplicon sequencing and marker-based genetic diversity analysis

For CAMK diversity analysis, publicly available sequences were retrieved from GenBank. In addition, between one to five CAMK amplicons per locality were sequenced from Philippine populations using either Sanger sequencing or the standard ONT amplicon sequencing protocol. The generated sequences were deposited in the NCBI GenBank under accession numbers PV232893.1 - PV232907.1, PV534023.1, PV694109.1 - PV694138.1 and PV448303 - PV448320. Additional samples collected in 2023 by collaborators in Lao PDR (Plant Protection Center), Cambodia (General Directorate of Agriculture), Vietnam (Plant Protection Research Institute), Thailand (Rayong Field Crops Research Center), and Indonesia (Universitas Muhammadiyah Surakarta) were also sequenced. A total of 155 CAMK gene sequences (>800 bp) were aligned using MAFFT with the Translation Align option implemented in Geneious Prime^®^ v2025.2.1 (Biomatters, New Zealand). The resulting alignment was manually inspected, and sequences showing extensive ambiguities or poor alignment quality were excluded prior to downstream analyses. Ambiguous positions were resolved by selecting the most frequent nucleotide at each variable site across the alignment. Haplotype relationships were inferred using a median-joining network implemented in PopART v1.7 (Leigh et al, 2015), with epsilon set to 0. Genetic diversity indices and neutrality tests were calculated using DnaSP v5 (Librado and Rozas, 2009). Other spatial and temporal haplotype trends were inspected using R v4.4.0 (R Core Team, 2023).

### 2.4 Fungal isolation and DNA extraction

Fungal isolation was performed following standard protocols for *C. theobromae* (Gil-Ordóñez et al., 2024; Pardo et al., 2024). Isolation was performed on 3–4 cm petiole sections, surface-sterilized immediately after field collection in 1% sodium hypochlorite for 1.5 minutes, followed by 2 minutes in 75% ethanol. Finally, the petioles were washed for 3 minutes using autoclaved distilled water and dried on sterile napkins before plated in water agar. Fungal growth was observed after 48 hours. From the emerging mycelia, 1 cm square was cut and transferred to potato dextrose agar (PDA) with corticium culture medium (CCM). Total DNA was isolated from 5-month-old colony of *C. theobromae* according with available protocols (Gil-Ordóñez et al., 2024; Pardo et al., 2024) with the following modifications: Using a needle loop scrape fungal mycelium 0.03 g and as precipitating agent we used ammonium acetate 3M instead of sodium acetate. Total DNA was quantified using the Qubit™ dsDNA HS Assay Kit (Invitrogen, Life Technologies, USA).

### 2.5 Genome sequencing, assembly, and analysis

Long read sequencing was performed using a PromethION 2 Solo (PS2) device. The sequencing library was prepared using ~800 ng of total DNA with SQK-LSK114 kit following the manufacturer’s instructions and processed on a R10.4.1 flow cell (FLO-PRO114M) for 72 h. Raw reads were aligned against the *C. theobromae* LAO1 hybrid reference genome (GCA_037974915.1) with Minimap2 v2.11 (Li, 2018), and reads not mapping the reference genome were discarded using SAMtools v1.3.1 (Li et al., 2009) to reduce assembly fragmentation and minimize the inclusion of low-complexity regions, duplicated haplotigs, and potential contaminants commonly observed in long-read-only assemblies. The filtered reads were then assembled *de novo* with Flye v2.9 (Kolmogorov et al., 2019) and contigs ≤1000 bp were filtered out. Genome analysis, *ab initio* gene prediction and ortholog comparison was performed as described in Gil-Ordóñez et al. (2024) using *C. theobormae* LAO1 (GenBank: GCA_037974915.1), *C. theobromae* CT2 (GenBank: GCA_009078325.1) and *Rhizoctonia solani* AG-1 IA (GenBank: GCA_016906535.1) genomes.

## 3 Results

### 3.1 Cassava witches’ broom symptoms were documented in all cassava-producing areas of the Philippines

**Figure 1:**
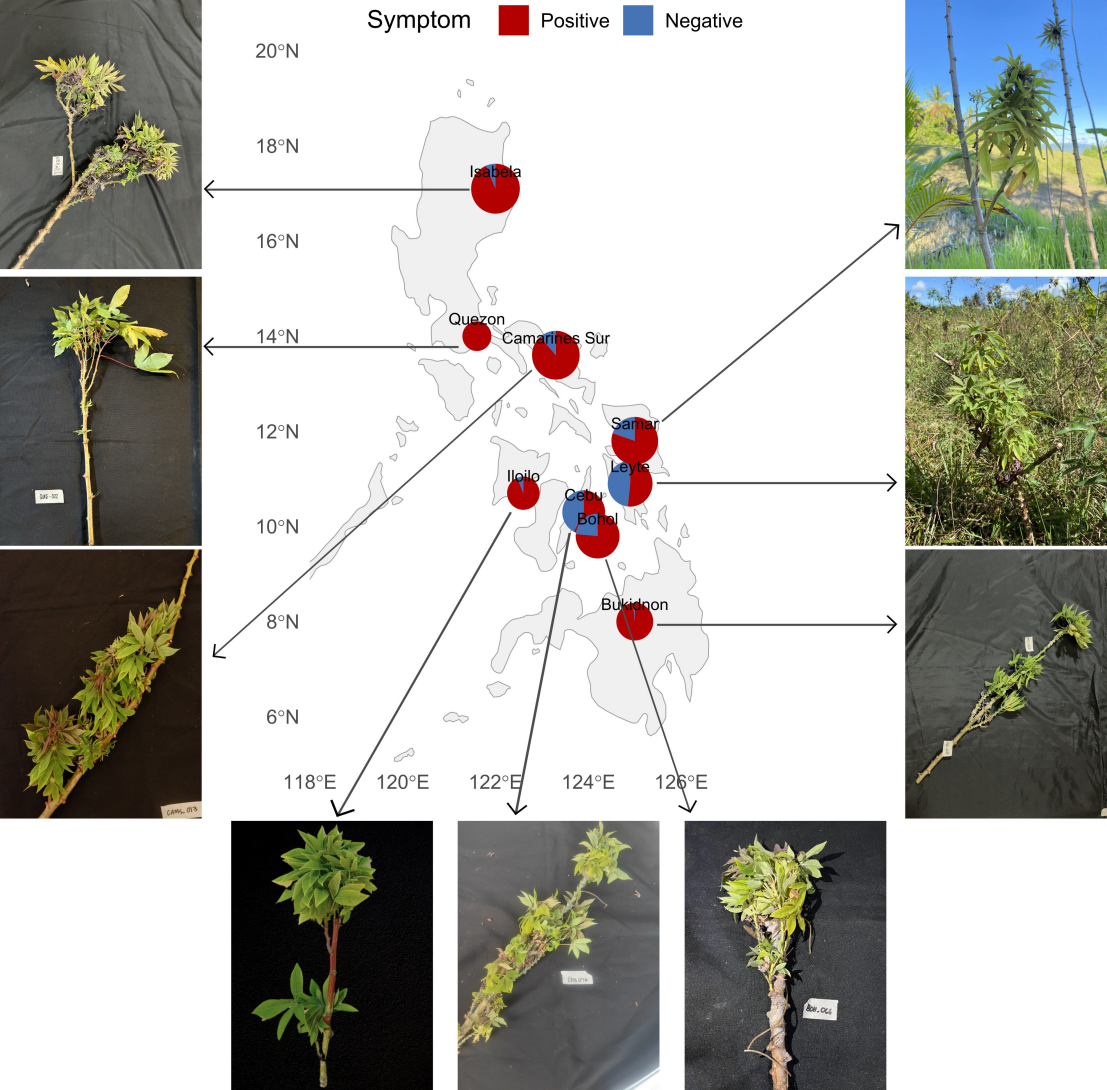
Geographic distribution and visual symptoms of cassava witches’ broom disease (CWBD) in the Philippines. A Philippine map showing the proportion of cassava plants displaying visual CWBD-like symptoms (“Positive”, red) or appearing asymptomatic (“Negative”, blue) in each surveyed province. The photographs represent the symptoms observed in the most common genotypes surveyed in each location (see **Supplementary Table 2**).

Plants exhibiting CWBD symptoms were observed in all visited provinces, but the incidence and severity of the symptoms varied per location (**Figure 1**). From a total of 735 observations, the highest incidences were found in the Luzon region (94.07 ± 5.73%) and Mindanao (98.41%), while the localities of the Visayas islands presented lower incidences in general (71.78 ± 17.17%). Image data collection also allowed us to confirm the absence of cassava mosaic disease (CMD) symptoms in all locations surveyed, an observation confirmed by PCR tests targeting SLCMV (**Supplementary Table 1**).

### 3.2 *C. theobromae* is associated with cassava witches’ broom in all surveyed provinces

A total of 567 CWBD samples (stems and petioles) were collected, of which, a representative subset (consisting of 211 samples) was used for the association study, where the first PCR screening identified 90 samples that were negative for the *C. theobromae* CAMK gene. To check whether the negative PCR results were influenced by the quality of the DNA extracted, we analyzed these samples using an internal control (*rbcLa* gene). We detected that 56 (or 26.54%) of these extracts did not amplify the internal control indicating that the quality of the DNA in these samples was not optimal and therefore they were not considered further in this analysis (**Supplementary Table 2**). We tested whether the excluded samples were distributed equally across provinces or whether they were associated with specific locations, using a binomial generalized linear model (GLM) at the sample level, which showed no significant association between locality and control PCR failure (p=0.89). Although the proportion of negative PCR controls varied across localities (Fisher’s exact test, p<0.001), this variation was likely influenced by differences in sample size among locations. In the end, 155 samples (134 petioles from symptomatic plants and 21 petioles from healthy plants) were tested for CAMK and used for the association analysis. This PCR diagnostic test for *C. theobromae* showed a sensitivity of 91.79% and a specificity of 95.24% (**Table 1**). By contrast, out of 141 samples tested for phytoplasma, only 28 produced a PCR band of the expected size, and although 27 of them originated from CWBD symptomatic plants, none of them corresponded to a phytoplasma sequence (**Supplementary Table 2**). Therefore, we found no evidence of a causal association between phytoplasma and CWBD.

**Table 1:** Results of PCR diagnostics using primers CIAT-CWBD-F2/R2 (Leiva et al., 2023).

Healthy	Nro of samples	PCR (-)	PCR (+)
Healthy	21	20	1
Diseased	134	11	123

A subgroup of PCR bands obtained were confirmed to correspond to *C. theobromae* through sequencing (**Supplementary Table S1**). The sensitivity (91.79%) was calculated as TP/(TP + FN)*100, and specificity (95.24%) calculated as TN/(TN + FP)*100. Confidence intervals were calculated using Wilson score method (95% confidence intervals) resulting in an interval from 85.9% to 95.3% for sensitivity and an interval of 77.3% to 99.1%, for specificity.TP, FN, TN, FP denotes True Negative, True Positive, False Negative, and False Positive, respectively.

### 3.3 Marker-based haplotype and genetic diversity analyses

Haplotype relationships at the CAMK locus were explored using a median-joining network based on 144 high-quality sequences, revealing a geographically structured population with centrally positioned and highly connected haplotypes in continental SEA, a peripheral cluster dominated by Philippine isolates in insular SEA, and American isolates clustering within continental SEA lineages (**Supplementary Figure 1**). Consistent with this topology, genetic diversity indices varied markedly among regions. Continental SEA exhibited the highest number of segregating sites and haplotypes (S = 43; H = 14), whereas the Americas were represented by a single haplotype with no polymorphism, supporting a recent introduction from this region. Despite harboring fewer haplotypes (H = 5), insular SEA displayed haplotype diversity comparable to that of the continental (Hd=0.659 vs. 0.679), indicating internal population structure likely influenced by limited sampling. Neutrality tests yielded non-significant values across regions (Tajima’s D and Fu and Li’s D/F), suggesting that the observed haplotype patterns are primarily shaped by demographic history rather than selection at the CAMK locus (**Table 2**).

**Table 2:** Genetic diversity indices and neutrality test results of *C. theobromae* calculated using DnaSP v5.

Origin	n	S	H	Hd	π (Pi)	k	Tajima’s D	Fu and Li’s D	Fu and Li’s F
Continental SEA	96	43	14	0,679	0,0059	5.007	-1.313 (p > 0.10)	0.872 (p > 0.10)	0.0105 (p > 0.10)
Insular SEA	40	9	5	0,659	0.0032	2.376	0.03128 (p > 0.10)	0.23920 (p > 0.10)	0.20295 (p > 0.10)
Americas	8	0	1	0	0	0	0	0	0
Total	144	43	16	0.809	0.0065	5.968	-0.752 (p > 0.10)	1.645 (p > 0.10)	0.767 (p > 0.10)

n, number of samples; S, number of polymorphic sites; H, haplotypes; Hd, haplotype diversity; π (Pi), nucleotide diversity; k, average number of nucleotide differences.

**Figure 2:**
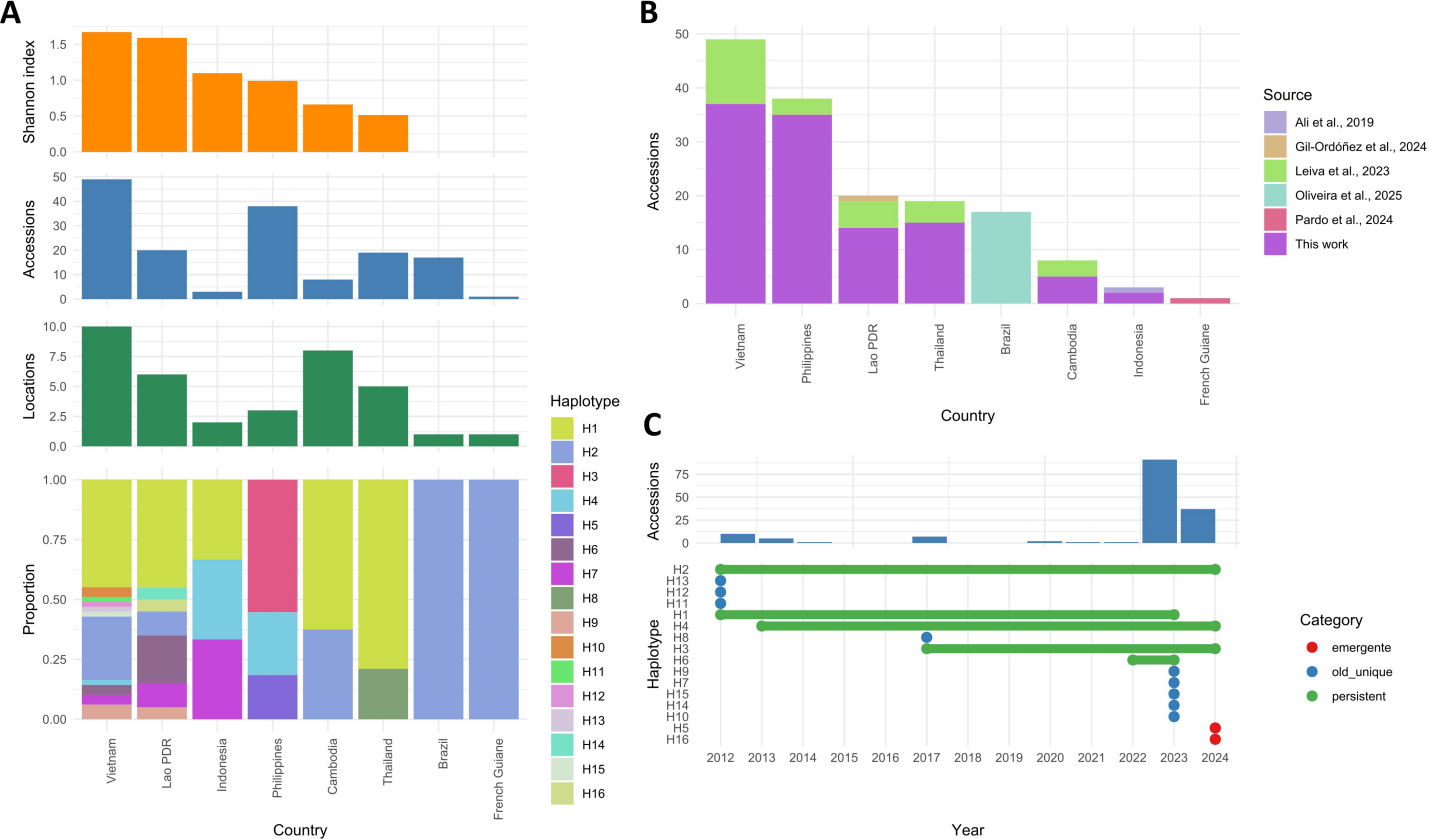
Geographic and temporal distribution of *C. theobromae* genetic haplotypes. **(A)** Diversity and composition of haplotypes by country, including the Shannon diversity index and the number of accessions per country. The haplotypes were numbered in descending order according to their frequency. **(B)** Contribution of different data sources to the overall dataset, highlighting the proportion of sequences generated in this study. **(C)** Temporal distribution of haplotypes from 2012 to 2024, showing persistent (green), newly emerged (red), and unique early (blue) haplotypes.

Based on haplotype distribution, Vietnam exhibited the highest genetic diversity, followed by the Philippines and Lao PDR, while Brazil and French Guiana showed the lowest values. Vietnam also contributed the largest number of accessions, whereas Indonesia and French Guiana were underrepresented. The most common haplotypes (H1, H2) were restricted to continental SEA, with H2 being the only haplotype detected in the Americas. Most remaining haplotypes were region-specific, with several exclusive to Vietnam, Lao PDR, or insular SEA, indicating a partial genetic connectivity between Asian subregions. Despite harboring fewer haplotypes, insular SEA displayed Shannon diversity values (>1) comparable to Vietnam and Lao PDR (>1.5), likely reflecting limited sampling and suggesting uncharacterized diversity (**Figure 2A**). Compared to the number of sequences retrieved from previous studies, those generated in this study substantially expanded the available dataset (**Figure 2B**). Temporal analysis (2012-2024) showed that most haplotypes persisted across multiple years, whereas a few emerged after 2021 or were restricted to early sampling periods, suggesting transient or geographically limited haplotypes (**Figure 2C**). Passport data are provided in **Supplementary Table 3**.

### 3.4 The genome of *C. theobromae* from the Philippines presents distinct characteristics

**Figure 3:**
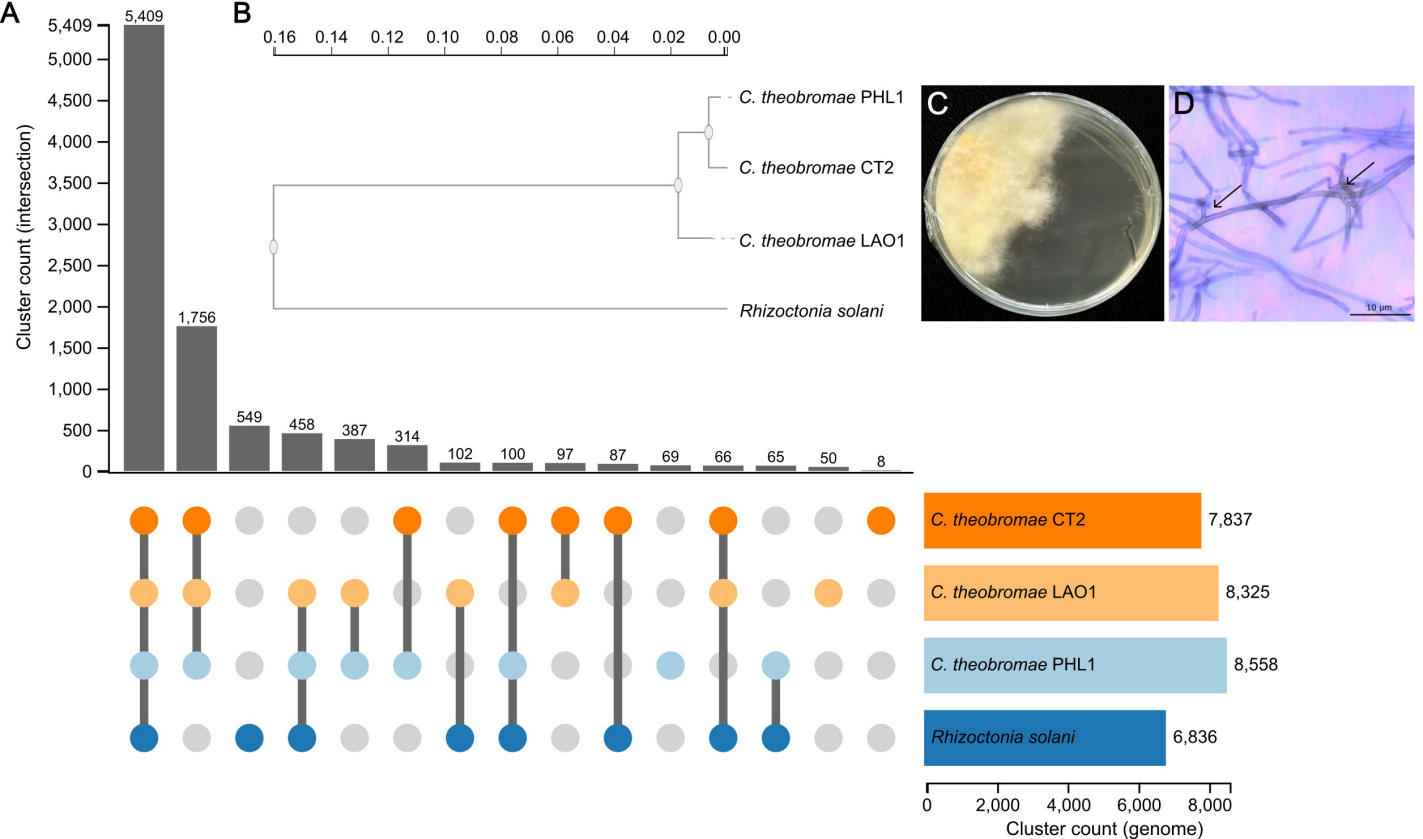
Comparative genomics of *C. theobromae* and *R. solani* isolates based on the content of orthologous gene clusters and their evolutionary relationship using a whole genome approach. **(A)** The UpSet diagram shows both unique and shared orthologous clusters between *C. theobromae* CT2 (dark orange dots), *C. theobromae* LAO1 (light orange dots), *C. theobromae* PHL1 (light blue dots) and (R) solani AG-1 IA (dark blue dots). On the left, a grayscale vertical bar graph indicates the number of shared orthologous clusters between species-specific combinations, represented by dark gray lines connecting the corresponding bars. On the right, a horizontal bar graph represents the total number of orthologous clusters identified in each strain: *C. theobromae* CT2 (dark orange bar), *C. theobromae* LAO1 (light orange bar), *C. theobromae* PHL1 (light blue bar), and *R. solani* AG-1 IA (blue bar). **(B)** Phylogeny of *C. theobromae* PHL1, *C. theobromae* CT2, *C. theobromae* LAO1 and *R. solani* AG-1 IA, inferred from conserved single-copy orthologous genes. The phylogenetic tree was constructed with Maximum Likelihood method, using the JTT + CAT evolutionary model and protein sequences. The SH test was used to assess the reliability of each node. **(C)** Five-month-old colony isolated from the petioles of symptomatic cassava plants from the province of Camarines Sur, Philippines. **(D)** Microscopic view with a magnification of 1000x. The presence of a septum is visible in each branched structure, which emerges at an almost right angle (black arrows).

Isolation of *C. theobromae* from various provinces was conducted following our previously published protocol (Gil-Ordóñez et al., 2024). The fungal pathogen was successfully isolated from symptomatic petioles, with hyphae emerging and growing from the ends of the petiole tissues onto the water agar. Single colonies were observed onto newly plated petri dishes to increase the amount of pure culture for DNA isolation (**Figure 3C**). DNA extraction produced 30 µg of DNA per petri dish. *De Novo* assembly of *C. theobromae* PHL1 generated an initial draft of 37,118,606 bp (733 contigs) prior to reference-based filtering. After filtering, 3.7% of the initial reads (3,037,185) were filtered out, and the genome assembly was reduced to 35,734,100 bp (average depth ~1,200X) in 528 contigs (N50 = 498,920 bp, L50 = 17 bp) with a G+C content of 48.64% (**Table 3**). *C. theobromae* belongs to the *Rhizoctonia* group and is a synonym of *Rhizoctonia theobromae*, therefore some entries could be annotated under both genera. Based on BLASTx results, 8 contigs (82,097 bp) of contaminants were discarded, with *Xanthomonas oryzae* being the most common contaminant (3 contigs, 10,942 bp). The remaining contigs were mainly classified as *C. theobromae* (244 contigs; 31.8 Mbp), followed by *Rhizoctonia* (60 contigs; 3.2 Mbp) and other species from Basidiomycota phylum (13 contigs; 165.6 Kbp) (**Supplementary Table 4**).

**Table 3:** Comparative statistics of *C. theobromae* genomes isolated from cacao in Indonesia (CT2), and from cassava in Lao PDR (LAO1) and the Philippines (PHL1).

Genome feature	*C. theobromae* CT2 (Ali et al., 2019)	*C. theobromae* LAO1 (Gil-Ordóñez et al., 2024)	*C. theobromae* PHL1 (this work)
Total length (bp)	33,899,105	33,386,600	35,734,100
Contig number	1,240	547	528
GC content (%)	49.22	48.93	48.64
Contig or scaffold N50	86,110	435,246	498,920
Contig or scaffold L50	87	18	17
Min contig length (bp)	200	1,000	1,000
Max contig length (bp)	589,277	2,528,383	2,484,883
Mean contig length (bp)	4,930	61,035.83	67,626.42
BUSCO Completeness (%)	99.10	94.50	97.20
Coverage (x) short reads	930	1,150.31	NA
Coverage (x) ONT reads	NA	12.04	1,227.00
Predicted genes	9,264	10,171	10,673
Gene density*^a^	0.59	0.52	0.52
Total gene length (bp)	20,074,964	17,435,730	18,595,116
Average gene length (bp)	2,168.07	1,714.26	1,742.26
Number of expressed genes*^b^	3,550 (38.32)	8,247 (81.08)	8,028 (75.22)

Values in parentheses indicate the relative frequency as a percentage of the total number of predicted genes.

*a CDS bases/total genome bases.

*b Only genes with sequence counts ≥10 detected in any of diseased cassava samples.

The contigs classified as *Rhizoctonia* likely reflect sequence homology with entries previously deposited under this nomenclature, rather than contamination by other *Rhizoctonia* species. This interpretation is supported by their coverage and patterns and GC content, which were consistent with the rest of the assembly, as well as the absence of taxonomic assignments to unrelated *Rhizoctonia* species. Accordingly, these contigs were considered part of the *C. theobromae* genome and retained in the final assembly. Based on the expected content of single copy orthologs from Eukaryota (eukaryota_odb10) and Agaricomycetes (agaricomycetes_odb10) assessed with BUSCO, the *C. theobromae* PHL1 genome has a completeness of 97.2% (complete: 93.3%, fragmented: 2.7%) (**Table 3**) and 86.9% (complete: 83.6%, fragmented: 3.3%), respectively. According to *ab initio* genetic prediction, *C. theobromae* PHL1 presents 10,673 genes with an average length of 1,742.26 bp (1,393.4 bp), of which 75.22% (8,028 genes) were validated by RNA-seq (**Table 3**). The final genome statistics are comparable with those of previously reported genomes for the species, with notable improvements in terms of contiguity (number of contigs, N50, L50) and completeness (assembly size, core genes, coverage) (**Table 3**).

According to the gene cluster analysis based on the predicted proteins of *C. theobromae* PHL1, LAO1, and CT2 isolates; and *R. solani* AG-1 IA, the fungal genomes share 5,409 orthologous gene clusters, with the highest association between *Ceratobasidium* isolates (1,756) and 69 singletons detected in isolate PHL1 (**Figure 3A**). Phylogenetic analysis based on the substitution model using the whole-genome approach revealed greater divergence between *R. solani* AG-1 IA and the *Ceratobasidium* isolate cluster (**Figure 3B**). Within the *Ceratobasidium* clade, CT2 and PHL1 formed the closest pair, showing the shortest branch distance between isolates. Based on this phylogenetic proximity, further functional characterization focused on the orthologous clusters shared exclusively between CT2 and PHL1. Among these, 314 orthologous clusters were shared exclusively between CT2 and PHL1. Gene ontology enrichment analysis of these clusters revealed significant overrepresentation of biological processes related to transmembrane transport (GO:0055085), polysaccharide catabolic process (GO:0000272), cellulose catabolic process (GO:0030245), response to antibiotic (GO:0046677), heme oxidation (GO:0006788), and Golgi calcium ion export (GO:0061454).

## 4 Discussion

CWBD has been long considered a phytoplasma-caused disease. In fact, there are several phytoplasma-related sequences of up to four ribosomal groups reported in NCBI GenBank originating from plants with CWBD symptoms (Pardo et al., 2023). Nevertheless, to this date, there is no concrete evidence of association of CWBD with phytoplasma – a situation largely believed to be a sampling problem caused by the unequal distribution and low titers of the pathogen within the cassava plant. On the other hand, results on CWBD in continental (continental) SEA recently indicated that the disease is rather associated with the fungus *C. theobromae* (Leiva et al., 2023). Similar results have been reported in French Guiana and Brazil where the disease has recently emerged (Pardo et al., 2024; Oliveira et al., 2025). Our molecular diagnostics results confirm that CWBD symptoms are not associated with SLCMV or phytoplasma.

Our surveys also confirm that CWBD is present in all cassava-growing regions of the Philippines with prevalence values above 50% at field level (**Figure 1**). Such widespread occurrence is consistent with that reported during national monitoring efforts between 2017 and 2019 (Dolores et al., 2023). As our field surveys were biased toward areas with previously reported disease presence, the prevalence values shown here cannot be considered representative at the regional scale. Additionally, in our diagnostic tests, *C. theobromae* was consistently associated with the characteristic CWBD symptoms with a specificity above 95% (**Table 1**), contrasting with the results obtained for phytoplasma, where none of the samples were true positives (**Supplementary Table 2**). This pattern is comparable to that reported in continental SEA (Leiva et al., 2023) and in a recent Philippine survey (Landicho et al., 2025). These results confirm the strong causal association between *C. theobromae* and CWBD and highlight that proper management of CWBD should target this fungal pathogen in all regions where the disease is present.

The geographically structured patterns inferred from CAMK are consistent with previous population-level analyses of *C. theobromae*, while extending their resolution. Using the same marker but under a more limited dataset, Oliveira et al. (2025) also reported weak but detectable geographic structuring and neutrality-consistent evolutionary dynamics, supporting a predominant role of isolation processes rather than adaptive divergence. Similarly, studies based on ribosomal markers (ITS and 28S) in *C. theobromae* identified extremely low sequence variation and clonal patterns, particularly amongst Philippine isolates (Landicho et al., 2025). Comparable levels of haplotype diversity have been reported in other fungal systems using the ITS region, where such variation has been interpreted as the result of neutral genetic variation structured by demographic history and geographic isolation (Merseguel et al., 2015; Olou et al., 2023). Under such scenarios, geographic structure may translate into asymmetric or directional patterns of dispersal when sampling spans multiple regions. In this context, haplotype diversity of CAMK is consistent with the historical reports of the disease, suggesting a spread from SEA to the Americas, with no evidence supporting the reverse movement. In addition, we did not detect SLCMV and no mosaic symptoms were observed in the Philippines, despite its widespread co-occurrence with CWBD in continental SEA (Siriwan et al., 2020). SLCMV and CMD have been reported in SEA since 2015 (Wang et al., 2016), occurring in all locations in SEA where CWBD was already present. The distinct *C. theobromae* CAMK haplotypes identified in the Philippines and the absence of CMD in this country may reflect asymmetries in the movement of planting materials (and inocula) between insular and continental regions.

The genome-level identity of *C. theobromae* as a fastidious fungus infecting cassava and cacao has been confirmed recently (Gil-Ordóñez et al., 2024). Although *in vitro* pure culture of *C. theobromae* is challenging, we succeeded in growing isolates from the Philippines and extract enough DNA to generate a refined reference genome with improved contiguity and gene annotation compared to previously available assemblies (Ali et al., 2019; Gil-Ordóñez et al., 2024). Results indicate that the Philippine isolate (PHL1) from cassava is more closely related to the isolate from cacao (CT2) than to continental SEA isolate (LAO1) from cassava, as can be observed in the complete genome comparisons (**Figure 3**). This close relationship is further supported by the 314 orthologous clusters shared exclusively between CT2 and PHL1, which likely represent a conserved functional core within these isolates. Enrichment analysis revealed overrepresentation of processes associated with transmembrane transport, ion homeostasis, and polysaccharide and cellulose catabolism, suggesting shared mechanisms for nutrient acquisition, plant cell wall degradation, and environmental adaptation (Kubicek et al., 2014). Additionally, enrichment of stress- and detoxification-related processes may reflect adaptation to competitive or stress-prone environments (Yaakoub et al., 2022). These observations also suggest that the recent introduction of *C. theobromae* to the Americas (Pardo et al., 2024; Oliveira et al., 2025), most likely originates from continental SEA (**Figure 2****;** **Supplementary Figure 1**).

While highly conserved markers such as CAMK, ITS, and 28S provide valuable baseline information and facilitate broad comparisons, their limited variability constraints fine-scale inference of population history and adaptive processes. Conversely, whole-genome approaches offer higher resolution but remain costly and logistically demanding, particularly for fastidious fungi. Future studies would therefore benefit from integrating intermediate-resolution strategies, such as targeted sequencing of variable genomic regions or the use of microsatellite markers, which have proven effective in resolving population structure in other fungi (Tsykun et al., 2017; Savva et al., 2025). Such approaches could bridge the gap between single-locus and genome-wide analyses, enabling a more robust reconstruction of the evolutionary history and dispersal routes *of C. theobromae*, particularly across insular regions such as Indonesia and Papua New Guinea where VSD of cacao is also present.

## Data Availability

The datasets generated for this study can be found in the online repositories. The names of the repository/repositories and accession number(s) can be found below: C. theobromae PHL1 genome BioProject: https://www.ncbi.nlm.nih.gov/bioproject/PRJNA1226245/, BioSample: https://www.ncbi.nlm.nih.gov/biosample/SAMN46924839/, SRA: https://www.ncbi.nlm.nih.gov/sra/SRX27798407, GenBank genome accession number: GCA_054508165.

## Supplementary material

Supplementary Figure 1 Median-joining haplotype network of *C. theobromae* based on the CAMK locus. Each circle represents a haplotype, with circle size proportional to the number of isolates. Colors indicate the geographic origin of isolates, shown by **(A)** geographic region and **(B)** by country. Small black circles represent inferred intermediate haplotypes not observed in the dataset, and hatch marks along branches indicate mutational steps separating haplotypes.
