# Supplementary figures and images for: Genome analysis of Ceratobasidium theobromae and its causal association with cassava witches’ broom disease in the Philippines
Source: Front Fungal Biol. 2026 Apr 17;7:1800255. doi: 10.3389/ffunb.2026.1800255 (PMC13132867; doi:10.3389/ffunb.2026.1800255)

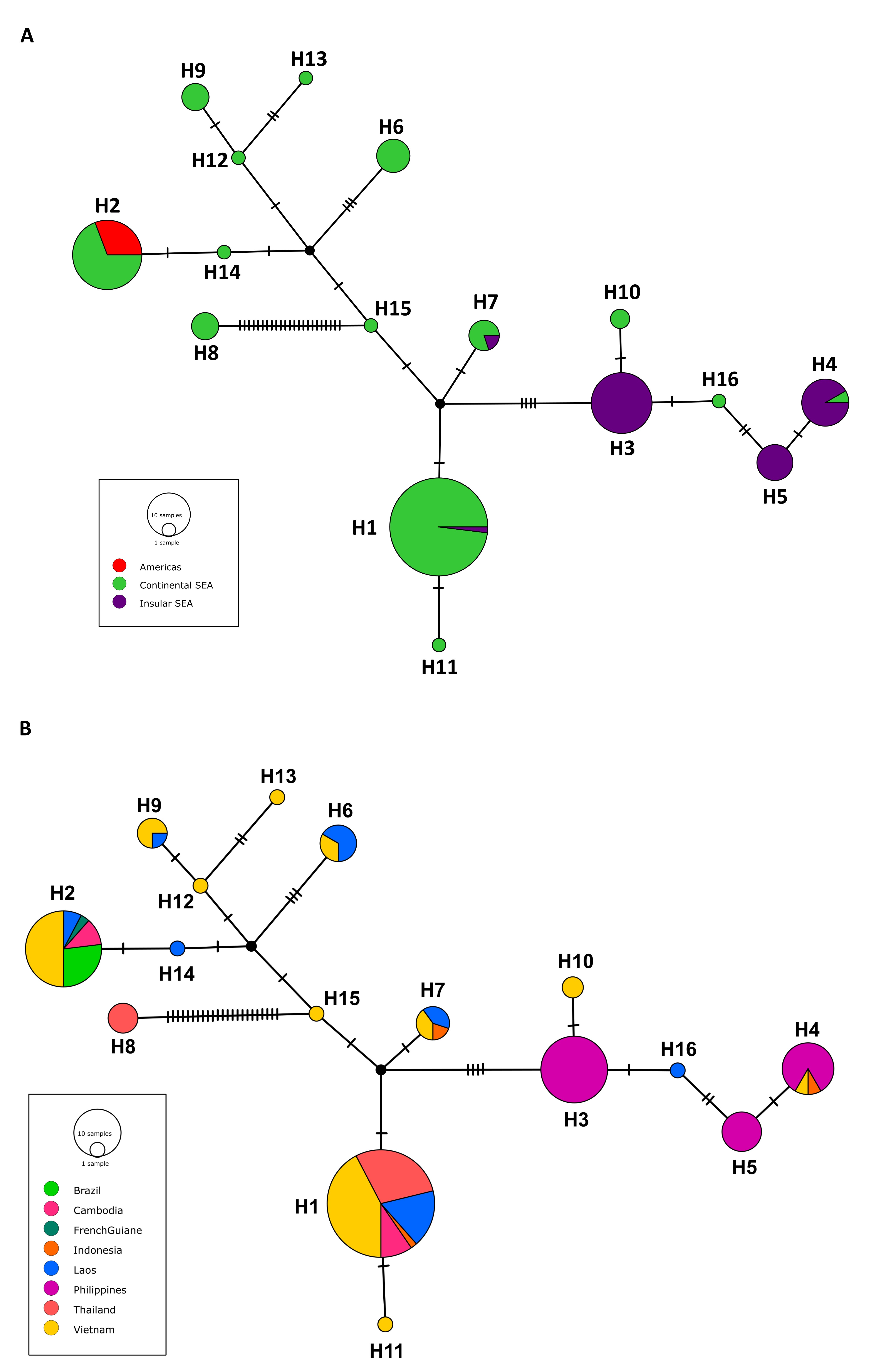

Supplement: Supplementary Figure 1 — Median-joining haplotype network of C. theobromae based on the CAMK locus. Each circle represents a haplotype, with circle size proportional to the number of isolates. Colors indicate the geographic origin of isolates, shown by (A) geographic region and (B) by country. Small black circles represent inferred intermediate haplotypes not observed in the dataset, and hatch marks along branches indicate mutational steps separating haplotypes. [file Image1.jpeg]
